# Microbial biotransformation to obtain stilbene methylglucoside with GPR119 agonistic activity

**DOI:** 10.3389/fmicb.2023.1148513

**Published:** 2023-03-22

**Authors:** Yu Peng, Yi Huan, Jing-Jing Chen, Tian-Jiao Chen, Lei Lei, Jin-Ling Yang, Zhu-Fang Shen, Ting Gong, Ping Zhu

**Affiliations:** State Key Laboratory of Bioactive Substance and Function of Natural Medicines, NHC Key Laboratory of Biosynthesis of Natural Products, CAMS Key Laboratory of Enzyme and Biocatalysis of Natural Drugs, Institute of Materia Medica, Chinese Academy of Medical Sciences and Peking Union Medical College, Beijing, China

**Keywords:** stilbenes, microbial transformation, *Beauveria bassiana*, methylglucosylation, GPR119 agonist, insulin secretion

## Abstract

**Introduction:**

Limitation of pharmaceutical application of resveratrol (RSV) and piceatannol (PIC) continue to exist, there is a need to obtain the superior analogs of two stilbenes with promoted activity, stability, and bioavailability. Microbial transformation has been suggested as a common and efficient strategy to solve the above problems.

**Methods:**

In this study, *Beauveria bassiana* was selected to transform RSV and PIC. LC-MS and NMR spectroscopies were used to analyze the transformed products and identify their structures. The biological activities of these metabolites were evaluated *in vitro* with GPR119 agonist and insulin secretion assays. Single factor tests were employed to optimize the biotransformation condition.

**Results:**

Three new methylglucosylated derivatives of PIC (**1**–**3**) and two known RSV methylglucosides (**4** and **5**) were isolated and characterized from the fermentation broth. Among them, **1** not only showed moderate GPR119 agonistic activity with 65.9%, but also promoted insulin secretion level significantly (12.94 ng/mg protein/hour) at 1 μM. After optimization of fermentation conditions, the yield of **1** reached 45.53%, which was increased by 4.2-fold compared with the control.

**Discussion:**

Our work presents that 3-*O*-MG PIC (**1**), obtained by microbial transformation, is an effective and safer ligand targeting GPR119, which lays a foundation for the anti-diabetic drug design in the future.

## 1. Introduction

Stilbenes have been proven to possess biological activities like anticancer (Filippis et al., 2017), anti-inflammatory (Dvorakova and Landa, 2017), neuroprotective (Chen et al., 2017), antibacterial (Singh A. et al., 2019), and antifungal properties (Filippis et al., 2019). Resveratrol (RSV) and its hydroxylated analog piceatannol (PIC), two of the best-known stilbenes, also closely resembled in various bioactivities (Kukreja et al., 2013, 2014; Banik et al., 2020). However, the low water solubility and bioavailability of either RSV or PIC limit their use in pharmaceutical and other fields (Delmas et al., 2011; Messiad et al., 2013). Therefore, it has great significance to obtain the diverse structural analogs of the two stilbenes for screening the lead compounds with improved activity, stability, and bioavailability.

Microbial transformation refers to the process of using microorganisms or their enzymes to convert substrates into structurally related products, which has long been used to produce natural product derivatives as a significant strategy (Venisetty and Ciddi, 2003). Compared to chemical synthesis, biotransformation has the characteristics including highly stereoselectivity and regioselectivity, mild reaction conditions, environmental friendliness, and uncomplicated experimental process, leading to the increasing body of information about the use of biocatalysis for selective transformations of synthetic and natural products (Eliwa et al., 2021). *Beauveria bassiana*, one of the best-known species of entomopathogenic fungi, is often used for microbial transformation due to its abilities to catalyze a wide variety of reactions such as hydroxylation (Xiong et al., 2006), reduction (Paula et al., 2015), Baeyer-Villiger oxidation (Świzdor et al., 2011), methylglucosylation (Eliwa et al., 2021), and acetylation (Zhan et al., 2010).

Type 2 diabetes mellitus (T2DM) accounts for nearly 90% of diabetes mellitus (DM) worldwide and is a chronic metabolic disorder characterized by hyperglycemia which is triggered by dysfunction of insulin secretion or insulin resistance (Tiwari et al., 2014; Manaithiya et al., 2021). Current clinical antidiabetic drugs, including sulfonylureas and glucagon-like peptide (GLP-1) receptor agonists, increase insulin secretion *via* different pharmacological mechanism. G protein-coupled receptor (GPR)119, predominantly expressed in the small intestine and islet *β*-cells, has been recognized as a promising therapeutic target for diabetes (Overton et al., 2008; Bahirat et al., 2017). Compared with other hypoglycemic drugs, GPR119 agonists have advantages of promoting both GLP-1 and insulin secretion in a glucose-dependent manner; but so far, no GPR119 candidate has successfully progressed into clinical application (Chu et al., 2006; Zhang et al., 2013; Xu et al., 2022). Novel strategies to discover GPR119 agonists need to be explored.

In this study, we selected *B. bassiana* as the biocatalyst to transform PIC and RSV. Five stilbene methylglucosides (**1**–**5**) were isolated and characterized from the transformed products, including three new ones (**1**–**3**). Then, we subjected the stilbene methylglucosides, together with RSV and PIC, to the active evaluation on the GPR119. Among them, 3-*O*-MG PIC (**1**) showed moderate GPR119 agonistic activity with 65.9%, and also significantly promoted insulin secretion level (12.94 ng/mg protein/h) at 1 μM. Finally, we optimized the biotransformation conditions to improve the yield of metabolite **1**, which laid the foundation for its structure modification and further hypoglycemic evaluation *in vivo*.

## 2. Materials and methods

### 2.1. General experimental procedures

Optical rotations and UV spectra were obtained on a JASCO P-2000 polarimeter and a JASCO V-650 spectrophotometer (JASCO Corporation, Tokyo, Japan), respectively. IR spectra were acquired by KBr disc method on a Shimadzu FTIR-8400S spectrometer (Shimadzu Co. Ltd., Tokyo, Japan). ^1^H NMR (500 MHz), ^13^C NMR (125 MHz), and HMBC spectra were run on Bruker AVIII-500 spectrometer with TMS (tetramethylsilane) as the internal standard (Bruker Biospin Corporation, Fallanden, Switzerland) in CD_3_OD. HRESIMS were performed on an Agilent 6520 HPLC-Q-TOF mass spectrometer (Agilent Technologies, Santa Clara, CA, United States). Semi-preparative HPLC was conducted on Agilent 1200 HPLC system (Agilent Technologies, Santa Clara, CA, United States) with a YMC-Pack ODS-A column (250 mm × 10 mm, 5 μm; YMC co., Ltd., Kyoto, Japan). The LC–MS experiments were accomplished by Shimadzu LCMS-2020 (Shimadzu Co. Ltd., Tokyo, Japan) equipped with a Shim-pack GIST C18 column (100 mm × 2.1 mm, 5 μm, Shimadzu Co. Ltd., Tokyo, Japan).

### 2.2. Strain and chemicals

The fungus *Beauveria bassiana* was purchased from China Agricultural Microorganism Culture Collection Center (ACCC 37297). Resveratrol (RSV, purity ≥ 98%) and piceatannol (PIC, purity ≥ 98%) were purchased from Shanghai Yuanye Bio-Technology Co., Ltd., China.

### 2.3. Biotransformation of RSV and PIC by *Beauveria bassiana*

*Beauveria bassiana* was cultured in a 500 mL flask with 100 mL liquid medium (2% glucose, 0.5% yeast extract, 0.5% tryptone, 0.5% NaCl, 0.5% KH_2_PO_4_, pH 6.4) and incubated at 28°C with shaking at 160 rpm for 2 days to obtain the seed culture. Each of the five identical 500-mL conical flasks with 90 mL fresh liquid medium and 15 mg of RSV dissolved in 250 μL DMSO was inoculated with 10 mL of the seed culture at 28°C with shaking at 160 rpm for 3 days. Another five identical 500-mL conical flasks with 90 mL fresh liquid medium and 15 mg of PIC dissolved in 250 μL DMSO were inoculated with 10 mL of the seed culture at the same conditions as described above. Culture controls consisted of fermentation blanks in which *B. bassiana* was grown under the same conditions but without the substrate. Substrate controls consisted of sterile medium containing the same amount of substrate under the same conditions but without the strain.

### 2.4. Analysis, isolation, and purification of metabolites

The culture was extracted twice with triple volume ethyl acetate, and ethyl acetate was removed under reduced pressure to yield a crude extract. The crude extract was redissolved in methanol for LC–MS analysis on a C18 column eluted (100 mm × 2.1 mm, 5 μm) with CH_3_CN-H_2_O (0 min-10%, 30 min-50%, 35 min-100%, 0.3 mL/min). The crude extract of PIC biotransformation products was further purified, using semi-preparative HPLC (CH_3_OH-H_2_O: 0 min-20%, 15 min-50%, 35 min-100%, 1.5 mL/min) equipped with the C18 column (250 × 10 mm, 5 μm) to yield compounds **1** (8 mg, 11%, *t*_R_ = 22 min), **2** (10 mg, 13%, *t*_R_ = 23 min), **3** (2.4 mg, 3%, *t*_R_ = 24.5 min). The crude extract of RSV biotransformation products was further purified using semi-preparative HPLC (CH_3_OH-H_2_O: 0 min-20%, 15 min-50%, 35 min-100%) to yield compounds **4** (26 mg, 35%, *t*_R_ = 23.5 min), and **5** (12 mg, 16%, *t*_R_ = 25.5 min).

#### 2.4.1. 3-*O*-(4″-*O*-methyl-*β*-D-glucopyranosyl)-piceatannol (3-*O-*MG PIC, **1**)

Brown amorphous powder; ${\left[\alpha \right]}_{D}^{20}$− 46.0 (c 0.1, CH_3_OH); IR *ν*_max_ 3,345, 1,601, 1,517, 1,449, 1,282, 1,081 cm^−1^ (Supplementary Figure S3); UV (CH_3_OH) λ_max_ (log ε) 202 (3.71), 217 (3.76), 303 (3.65), 326 (3.76) nm (Supplementary Figure S2); ^1^H NMR (CD_3_OD, 500 MHz) and ^13^C NMR (CD_3_OD, 125 MHz) data, see Table 1 and Supplementary Figures S4-S6; HRESIMS: *m/z* 421.1485 [M + H]^+^ (calcd. For C_21_H_25_O_9_, 421.1493) (Supplementary Figure S1).

**Table 1:** ^1^H (500 MHz) and ^13^C NMR (125 MHz) spectral data of metabolites **1–3** in CD_3_OD.

Position	1		2		3	
	*δ*_H_ (*J* in Hz)	*δ*_C_	*δ*_H_ (*J* in Hz)	*δ*_C_	*δ*_H_ (*J* in Hz)	*δ*_C_
1		140.0		139.5		139.7
2	6.77, t (2)	105.6	6.47, d (2)	104.6	6.46, d (2)	104.4
3		159.0		158.3		158.3
4	6.45, t (2)	102.6	6.19, t (2)	102.5	6.17, t (2)	103
5		158.2		158.3		158.3
6	6.62, t (2)	106.8	6.47, d (2)	104.6	6.46, d (2)	104.4
7	6.96, d (16)	125.2	6.85, d (16)	127.4	6.85, d (16)	126.5
8	6.81, d (16)	128.9	6.94, d (16)	127.6	6.95, d (16)	127.7
1′		129.5		133.2		129.9
2′	7.00, d (2)	112.5	7.05, d (2)	113.1	7.44, d (2)	115.2
3′		145.1		145.1		146.9
4′		145.3		147.0		145.6
5′	6.75, d (8.5)	115.0	7.16, d (8.5)	117.1	6.83, d (8.5)	115.8
6′	6.86, dd (8.5, 2)	118.9	6.96, dd (8.5, 2)	118.0	7.09, dd (8.5, 2)	122.2
1″	4.89, d (8)	100.8	4.85, d (8)	101.6	4.81, d (8)	101.3
2″	3.47, dd (9, 8)	73.6	3.53, dd (9, 8)	73.5	3.54, dd (9, 8)	73.6
3″	3.58, d (9)	76.6	3.59, d (9)	76.1	3.62, d (9)	76.2
4″	3.21, t (9)	79.3	3.24, t (9)	79.1	3.21, t (9)	79.4
5″	3.45, m	75.8	3.43, m	75.8	3.49, m	76.0
6″	3.90, dd (12, 2)	60.7	3.88, dd (12, 2)	60.5	3.92, dd (12, 2)	60.7
	3.74, dd (12, 5)		3.75, dd (12, 5)		3.76, dd (12, 5)	
4″-*O-*CH_3_	3.59, s	59.5	3.62, s	59.6	3.62, s	59.5

#### 2.4.2. 4′-*O*-(4″-*O*-methyl-*β*-D-glucopyranosyl)-piceatannol (4′-*O-*MG PIC, **2**)

Brown amorphous powder; ${\left[\alpha \right]}_{D}^{20}$− 32.0 (c 0.1, CH_3_OH); IR *ν*_max_ 3,388, 1,646, 1,596, 1,509, 1,444, 1,261, and 1,078 cm^−1^ (Supplementary Figure S9); UV (CH_3_OH) λ_max_ (log ε) 203 (3.88), 216 (3.80), 302 (3.70), and 319 (3.75) nm (Supplementary Figure S8); ^1^H NMR (CD_3_OD, 500 MHz); and ^13^C NMR (CD_3_OD, 125 MHz) data, see Table 1 and Supplementary Figures S10-S12; HRESIMS: *m/z* 421.1484 [M + H]^+^ (calcd. For C_21_H_25_O_9_, 421.1493) (Supplementary Figure S7).

#### 2.4.3. 3′-*O*-(4″-*O*-methyl-*β*-D-glucopyranosyl)-piceatannol (3′-*O-*MG PIC, **3**)

Brown amorphous powder; ${\left[\alpha \right]}_{D}^{20}$− 16.0 (c 0.1, CH_3_OH); IR *ν*_max_ 3,223, 1,662, 1,593, 1,514, 1,443, 1,167, and 1,049 cm^−1^ (Supplementary Figure S15); UV (CH_3_OH) λ_max_ (log ε) 204 (3.68), 218 (3.67), 239 (3.59), 302 (3.60), and 320 (3.64) nm (Supplementary Figure S14); ^1^H NMR (CD_3_OD, 500 MHz); and ^13^C NMR (CD_3_OD, 125 MHz) data, see Table 1 and Supplementary Figures S16-S18; HRESIMS: *m/z* 421.1485 [M + H]^+^ (calcd. For C_21_H_25_O_9_, 421.1493) (Supplementary Figure S13).

#### 2.4.4. 4′-*O*-(4″-*O*-methyl-*β*-D-glucopyranosyl)-resveratrol (4′-*O-*MG RSV, **4**)

Light yellow amorphous powder; 1H NMR data (CD3OD, 500 MHz): 7.47 (1H, d, *J* = 9 Hz, H-2′/6′), 7.08 (1H, d, *J* = 9 Hz, H-3′/5′), 7.01 (1H, d, *J* = 16 Hz, H-7), 6.90 (1H, d, *J* = 16 Hz, H-8), 6.48 (1H, t, *J* = 2 Hz, H-2/6), 6.19 (1H, t, *J* = 2 Hz, H-4), 4.92 (1H, d, *J* = 7.5 Hz, H-1″), 3.87 (1H, dd, *J* = 12, 2 Hz, H-6″), 3.74 (1H, dd, *J* = 12, 5 Hz, H-6″), 3.61 (3H, s, 4″-OCH_3_), 3.58 (1H, d, *J* = 9 Hz, H-3″), 3.48 (1H, dd, *J* = 9.5, 8 Hz, H-2″), 3.45 (1H, m, H-5″), 3.22 (1H, t, *J* = 10 Hz, H-4″) (Supplementary Figure S19). ESIMS: *m/z* 403.25 [M−H]^−^.

#### 2.4.5. 3-*O*-(4″-*O*-methyl-*β*-D-glucopyranosyl)-resveratrol (3-*O-*MG RSV, **5**)

Light yellow amorphous powder; ^1^H NMR data (CD_3_OD, 500 MHz): 7.39 (1H, d, *J* = 9 Hz, H-2′/6′), 7.03 (1H, d, *J* = 16 Hz, H-7), 6.86 (1H, d, *J* = 16 Hz, H-8), 6.78 (1H, t, *J* = 2 Hz, H-2), 6.78 (1H, d, *J* = 9 Hz, H-3′/5′), 6.63 (1H, t, *J* = 2 Hz, H-6), 6.45 (1H, t, *J* = 2 Hz, H-4), 4.89 (1H, d, *J* = 7.5 Hz, H-1″), 3.86 (1H, dd, *J* = 12, 2 Hz, H-6″), 3.74 (1H, dd, *J* = 12, 5 Hz, H-6″), 3.58 (1H, d, *J* = 9 Hz, H-3″), 3.62 (3H, s, 4″-OCH_3_), 3.47 (1H, dd, *J* = 9.5, 8 Hz, H-2″) 3.46 (1H, m, H-5″), and 3.21 (1H, t, *J* = 10 Hz, H-4″) (Supplementary Figure S20). ESIMS: *m/z* 403.25 [M−H]^−^.

### 2.5. GPR119 agonist and glucose-stimulated insulin secretion assay

To detect potential transactivation of the indicated compound to GPR119, 293 T cells were transiently transfected using Lipofectamine 2000 with plasmids constructions mentioned in previous work (Lei et al., 2015), following treated with compounds at designated concentrations and DMSO as vehicle, then chemiluminescence value reflecting to luciferase activity was measured by a microplate reader using the firefly-luciferase assay kit. The activation fold was calculated as value compound/value vehicle, and relative activity percentage of indicated compounds was normalized to the activation fold of positive control, APD597.

The mouse pancreatic *β*-cell line MIN6 were used to evaluate insulin secretion in response to high concentrations of glucose (Lei et al., 2015). MIN6 cells were seeded in 96-well plates with Dulbecco’s modified Eagle’s medium (DMEM, 25 mM glucose) supplemented with 15% fetal bovine blood (FBS). Before the glucose-stimulated insulin secretion test, the cells were starved for 1 h in Krebs buffer (2.8 mM glucose) with or without 1 μM compound **1** or APD. Then, the cells were incubated for another 1 h in new Krebs containing 16.8 mM glucose combined with compound **1**, APD, or vehicle. The supernatant was collected for the insulin test *via* the Mouse Ultrasensitive Insulin ELISA Kit (80-INSMSU-E10, ALPCO, Salem, NH, United States), and MIN6 cell lysates were used for protein quantitative determination with BCA reagents (P1511; APPLYGEN, Beijing, China).

All experiments were performed in triplicate. When continuous data were normality distributed, it was shown as Mean ± standard error of the mean (SEM) and determined with one-way ANOVA test. Calculations were performed with Prism software. Statistical significance was taken at *p < 0.05, **p < 0.01, ***p < 0.001.

### 2.6. Optimization of the biotransformation conditions to improve the yield of metabolite 1

Different pH of liquid medium: *B. bassiana* was cultured in three 100-mL flasks, each containing 20 mL of liquid medium with pH 6.5, 7.0, and 7.5, respectively, and incubated at 28°C, 160 rpm for 2 days to obtain the seed culture. 2 mL of each seed culture was inoculated in the 100-mL conical flask containing 3 mg of PIC dissolved in 50 μL DMSO and 18 mL of fresh liquid medium with the corresponding pH 6.5, 7.0, and 7.5, respectively, and incubated at the aforementioned conditions for 3 days.

#### 2.6.1. Different temperature

The culture process was the same as that in the pH optimization including the temperature was set at 28°C for the initial cultivation, except that the pH was set at 7.0 for the whole process and the temperatures were set at 24°C, 28°C, 32°C, and 37°C, respectively, at the transformation stage.

#### 2.6.2. Different shaking speed

The culture process was the same as that in the temperature optimization including the pH was set at 7.0 for the whole process, except that the temperature was set at 32°C for the whole process and the shaking speeds were set at 160, 190, and 220 rpm, respectively, for the whole process.

#### 2.6.3. Different culture time

The culture process was the same as that in the shaking speed optimization except that the shaking speed was set at 190 rpm and the transformation was conducted for 2, 3, 4, and 5 days, respectively.

All the above biotransformation products were tested and analyzed according to the method described in section 2.4. The data were presented as means ± SEM (*n* = 3).

## 3. Results and discussion

### 3.1. Biotransformation of PIC and RSV by *Beauveria bassiana*

**Figure 1:**
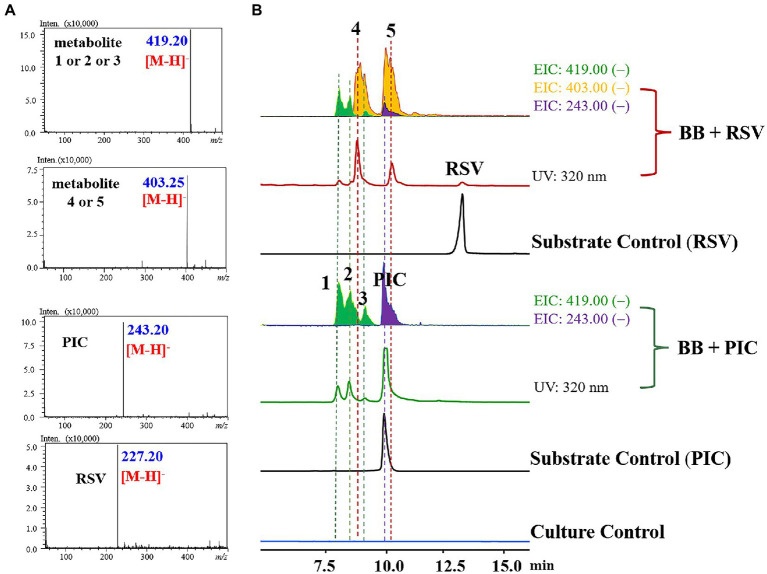
HPLC–MS results for the biotransformation products of RSV and PIC by *Beauveria bassiana* (**A**: MS data; **B**: LC–MS profile). BB + RSV or BB + PIC group infers EIC analysis and HPLC chromatogram of the products of RSV or PIC transformed by *B. bassiana* for 3 days; Substrate control consisted of sterile medium containing the same amount of substrate under the same conditions but without the strain; Culture control consisted of fermentation blanks in which the strain was grown under the same conditions but without the substrate.

To investigate the biotransformation capability of *B. bassiana* on RSV and PIC, the two substrates were, respectively, incubated in the culture medium with *B. bassiana* for 3 days. Then, the extracts of the culture medium were analyzed using HPLC-UV/MS (Figure 1).

**Figure 2:**
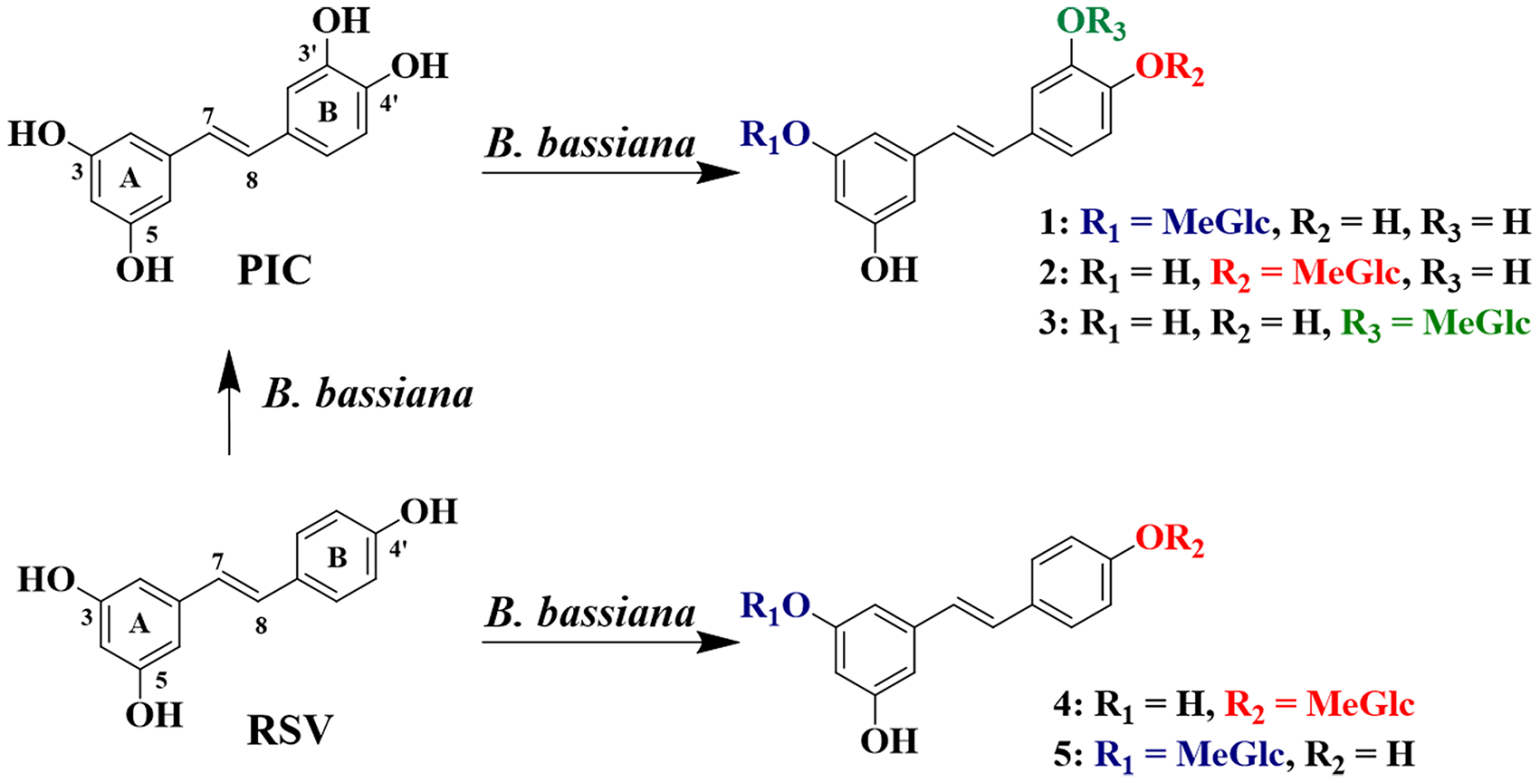
Conversion route of RSV and PIC by *Beauveria bassiana.*

The LC–MS results revealed that the three major metabolites in PIC group all exhibited [M − H]^−^ at *m/z* 419.20, with an increase of 172 a.m.u. over PIC, turning out the introduction of *O*-methyl-glucose. Three minor metabolites (**1**–**3**, *m/z* 419.20 [M − H]^−^), two major ones (**4** and **5**, *m/z* 403.25 [M − H]^−^), and a trace amount of PIC (*m/z* 243.20 [M − H]^−^) were detected in RSV group, indicating that *B. bassiana* could not only *O*-methylglucosylate RSV, but also hydroxylate it at C-3′. To our knowledge, this is the first report that *B. bassiana* has the capability to hydroxylate RSV. In PIC group, *B. bassiana* also exhibited the *O*-methylglucosylation activity and three metabolites (**1**–**3**) with the [M − H]^−^ ion at *m/z* 419.20 were detected (Figures 1, 2).

Metabolites **1**–**5** were then prepared on a larger scale for structural identification. Metabolites **4** and **5** from RSV group were identified as 4′-*O*-(4″-*O*-methyl-*β*-D-glucopyranosyl)-resveratrol (4′-*O-*MG RSV, **4**) and 3-*O*-(4″-*O*-methyl-*β*-D-glucopyranosyl)-resveratrol (3-*O-*MG RSV, **5**) [16, 23] by comparing their spectroscopic data with those of the reported structures. Metabolites **1–3** from PIC group were identified as 3-*O*-(4″-*O*-methyl-*β*-D-glucopyranosyl)-piceatannol (3-*O-*MG PIC, **1**), 4′-*O*-(4″-*O*-methyl-*β*-D-glucopyranosyl)-piceatannol (4′-*O-*MG PIC, **2**), and 3′-*O*-(4″-*O*-methyl-*β*-D-glucopyranosyl)-piceatannol (3′-*O-*MG PIC, **3**) by HRESIMS, 1D NMR, and 2D NMR spectra.

### 3.2. Structural elucidation of metabolites 1–3

**Figure 3:**
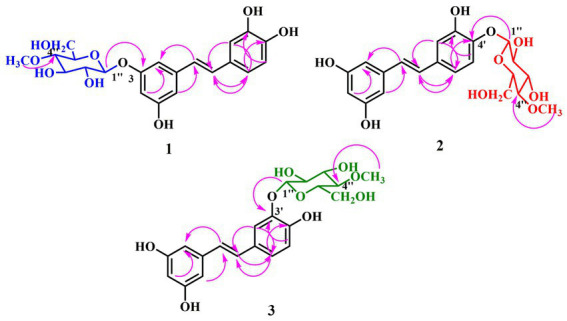
Key HMBC correlations of metabolites **1**–**3**.

Metabolite **1** was obtained as brown amorphous powder. The HRESIMS exhibited a molecular ion at *m/z* 421.1485 [M + H]^+^ (Supplementary Figure S1), suggesting the molecular formula C_21_H_24_O_9_ with an increase of 172 a.m.u. over PIC, which was proof of the introduction of *O*-methyl-glucose. In the ^1^H-NMR spectrum (Table 1), the methoxy signal at *δ* 3.59 (3H, s) and other hydrogen signals at *δ* 4.89 (1H, d, *J* = 8 Hz), *δ* 3.74 (1H, dd, *J* = 5, 12 Hz), *δ* 3.58 (1H, d, *J* = 9 Hz), *δ* 3.47 (1H, dd, *J* = 8, 9 Hz), *δ* 3.90 (1H, dd, *J* = 2, 12 Hz), *δ* 3.45 (1H, m), and *δ* 3.21 (1H, t, *J* = 9 Hz) were basically consistent with the chemical shift values of 4″-*O*-methyl-glucose moiety (Zhan et al., 2010). Moreover, the typical carbon signals of 4″-*O*-methyl-glucose unit at *δ* 100.8, 73.6, 76.6, 79.3, 75.8, 60.7, and 59.5 were found in ^13^C-NMR spectrum (Table 1, Supplementary Figure S5; Zhan et al., 2010). The anomeric proton at *δ* 4.89 (1H, d, *J* = 8.0 Hz) interacted with the C-3 carbon signal at *δ* 159.0 in the HMBC spectrum (Figure 3, Supplementary Figure S6), indicating that the glucosyl unit was introduced at C-3 position. In addition, C-2 and C-3 are in the equal chemical environment in PIC, resulting that the chemical shift values of the two carbons are the same. However, the two different chemical shift signals at *δ* 105.6 and 106.8 in ^13^C-NMR spectrum of compound **1** (Supplementary Figure S5) further confirmed that the 4″-*O*-methyl-glucose moiety was introduced into A-ring of PIC. A large coupling constant (*J* = 8.0 Hz) was observed for the anomeric proton, confirming the *β*-configuration of the glucosidic bonds. The rest of the ^1^H and ^13^C-NMR signals resembled with PIC. In summary, compound **1** was identified as 3-*O*-(4″-*O*-methyl-*β*-D-glucopyranosyl)-piceatannol (3-*O-*MG PIC, Figure 3).

Metabolites **2** and **3** were purified as brown amorphous powders. Their molecular formulas were deduced as C_21_H_24_O_9_ from HRESIMS (*m/z* 421.1484 and 421.1485, respectively [M + H]^+^) (Supplementary Figures S7, S13), suggesting that they might be the isomers of **1**. Detailed analysis of their ^1^H and ^13^C NMR spectra (Table 1) showed that 4″-*O*-methylglucopyranosides were connected to B-rings of PIC, due to the remaining signals bore resemblance to those of PIC, especially A-rings. The sugar unit of metabolite **2** should be connected to C-4′ position, which was supported by the HMBC correlation between the anomeric proton at *δ* 4.85 (d, *J* = 8.0 Hz) and C-4′ (*δ* 147.0; Figure 3, Supplementary Figure S12). Moreover, the evident downfield shifts of C-4′ (*δ* 147.0) and its para-position carbon (*δ* 133.2) confirmed the substitution position of sugar unit of **2** (Supplementary Figure S11). On the other hand, the 4″-*O*-methylglucopyranose fragment was attached to C-3′ of PIC in metabolite **3** as illustrated by the HMBC correlation between H-1″ (*δ* 4.81, d, *J* = 7.5 Hz) and C-3′ (*δ* 146.9) (Supplementary Figure S18). The large *J*-values of coupling constants of H-1″ in metabolites **2** and **3** were indicative of the *β*-oriented anomeric protons (Figure 3, Table 1). On the basis of the above evidences, the structures of **2** and **3** were determined to be 4′-*O*-(4″-*O*-methyl-*β*-D-glucopyranosyl)-piceatannol (4′-*O-*MG PIC, **2**) and 3′-*O*-(4″-*O*-methyl-*β*-D-glucopyranosyl)-piceatannol (3′-*O-*MG PIC, **3**).

### 3.3. GPR119 agonist and insulin secretion assay

**Figure 4:**
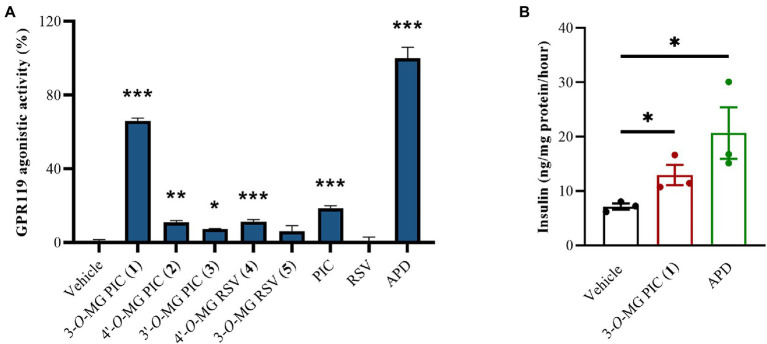
**(A)** GPR119 agonistic activity of two stilbene aglycones (PIC and RSV) and the methylglucosylated derivatives of them (**1**–**5**) at 1 μM *in vitro*. **(B)** The effect of **1** on glucose-stimulated insulin secretion. The mouse pancreatic *β*-cell line MIN6 were cultured in Krebs buffer (containing 16.8 mM glucose) with or without **1** or APD at 1 μM for 1 h. Vehicle refers to saline. APD refers to the positive control. All data were expressed as the mean ± SEM (*n* = 3). *p < 0.05, **p < 0.01, ***p < 0.001, vs. vehicle group. Data were analyzed by the one-way ANOVA test.

Two stilbene aglycones (PIC and RSV) and their methylglucosides (**1**–**5**) were screened for the agonistic activity of GPR119 at the concentration of 1 μM *in vitro*. As shown in Figure 4A, 3-*O*-MG RSV (**4**) and 4′-*O*-MG RSV (**5**) exhibited slightly lower GPR119 agonistic activity than RSV (0.7%), and the relative activity rates were 11.5% and 6.2%, respectively. In contrast, metabolite **1**, the methylglucosylated derivative of PIC, showed activity of 65.9%, which was more active than its aglycone with activity of 18.5%. Apparently, the methylglucosylation at 3-OH is superior to 3′ or 4′-OH for the improvement of agonistic activity of PIC, as evidenced by **1** vs. **2** and **3**. Next, with the aim of further exploring the effects of compound **1** on glucose-stimulated insulin secretion, we tested it in mouse pancreatic *β*-cell line MIN6. At a high concentration of glucose (16.8 mM), insulin secretion level (12.94 ng/mg protein/h) was significantly increased by compound **1** in MIN6 cells (Figure 4B). The above results indicated that 3-*O*-MG PIC (**1**) could ameliorate pancreatic *β*-cell function and could be a potential new candidate as a ligand of GPR119 for the treatment of type 2 diabetes.

### 3.4. Optimization of the biotransformation conditions to improve the yield of metabolite 1

In order to increase the yield of metabolite **1**, we optimized the fermentation conditions for biotransformation of PIC by *B bassiana*. The effects of different pH of medium (6.5, 7.0, and 7.5), culture temperature (24°C, 28°C, 32°C, and 37°C), rotational speed (160, 190, and 220 rpm), and culture duration (2, 3, 4, and 5 days) on the yield of compound **1** were determined, to investigate the optimal fermentation conditions.

**Figure 5:**
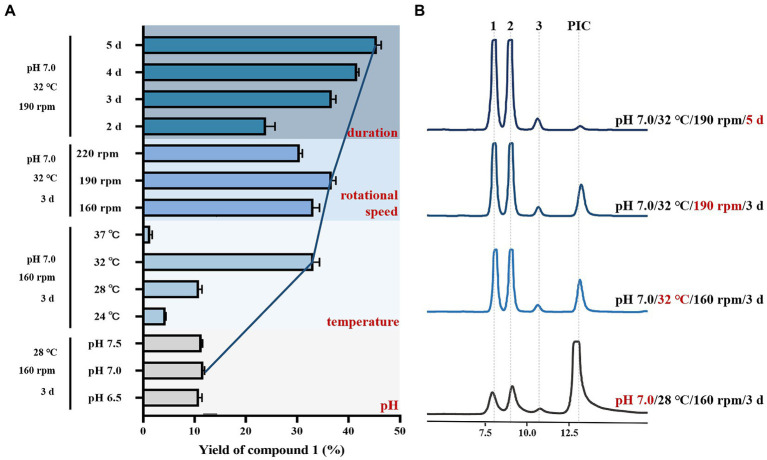
Optimization of the fermentation conditions for biotransformation of PIC by *Beauveria bassiana*. **(A)** Yield of **1** under different fermentation conditions, and the data represent the mean ± SEM, *n* = 3. **(B)** HPLC chromatograms of crude extract of biotransformation broth.

Based on the initial fermentation conditions (pH 6.5, 28°C, 160 rpm, 3 days), we first tested the effect of different pH (6.5, 7.0, 7.5) of medium on the yield of **1**. The fermentation system had a certain tolerance to pH, causing that the yield of **1** was not much different in the pH range from 6.5 to 7.5. However, according to the results, it can still be concluded that the biotransformation yield (11.7%) of **1** by *B bassiana* is the highest when pH of liquid medium was 7.0 (Figure 5). Next, the effects of different temperatures were tested based on changing the pH condition (pH 7.0, 160 rpm, 3 days). The results verified that the bioconversion yield of **1** increased gradually with the increase of temperature, and reached the highest (33.17%) at 32°C. Nevertheless, the growth of *B. bassiana* was inhibited on account of high temperature at 37°C, resulting that the biotransformation process hardly occurred (Figure 5). Additionally, the biotransformation yield of **1** changed inconspicuously with different rotational speed (Figure 5). Finally, the effects of different incubation time on the yield were investigated with the improved fermentation conditions (pH 7.0, 32°C, 190 rpm). With the extension of culture duration, the bioconversion yield of **1** increased, which reached 45.53% on the 5th day of culturing when almost no PIC was left (Figure 5).

Above single factor test showed that the strength of impact of the various factors on the yield of **1** was in the order temperature > culture duration > rational speed > pH of medium. The yield of metabolite **1** reached 45.53% which was increased by 4.2-fold under the optimized fermentation conditions of pH 7.0, fermentation temperature 32°C, rotational speed 190 rpm, and the culture time 5 days, when the substrate concentration was 0.15 mg/mL.

## 4. Discussion

Glycosylation is an effective strategy to improve physicochemical and biological properties of nature products. Interestingly, special glucosides of some substances, with unique sugar unit, 4-*O*-methyl-D-glucopyranose, were identified through biotransformation methods by *B. bassiana* (Zhan and Gunatilaka, 2006; Zhan and Gunatilaka, 2008; Ha et al., 2021). Compared with common sugar conjugates, methylglucosides displayed increased stability against glycoside hydrolysis without losing aqueous solubility (Xie et al., 2019). A set of glycosyltransferase-methyltransferase (GT-MT) gene pairs that encode the methylglucosylation function were uncovered from the fungus *B. bassiana* and other hypocreales fungi, followed by the heterologous expression in *Saccharomyces cerevisiae* system. These GT-MT modules displayed decent substrate promiscuity and suitable regiospecificity, methylglucosylating a panel of natural products such as flavonoids, stilbenoids, anthraquinones, and benzenediol lactones (Xie et al., 2018, 2019). In order to obtain the superior RSV and PIC metabolites with enhanced efficiency and safety, we used *B. bassiana* to biotransform these two substances. To our knowledge, we probed the biotransformation of PIC by *B. bassiana* for the first time, obtaining three novel PIC mono-methylglucosides (**1**–**3**). Similar to the case of RSV, 4′-OH of phenolic ring of PIC was favor for methylglucosylation to yield metabolite **2**, but the difference is that the proportion of 3-*O*-MG PIC (**1**) was raised, which may be due to the reduction of the yield of **2** caused by steric hindrance of 3′-OH. In addition, it is worth noting that the minor PIC methylglucosides were detected in RSV case, revealing that the type of reactions catalyzed by *B. bassiana* also included the hydroxylation of the benzene ring, which has not been explored as of yet, and further genome mining and heterologous expression efforts will disclose the hydroxylase with this function in *B. bassiana*.

T2DM presents with a spectrum of dysfunctions, characterized by hyperglycemia, insulin resistance and consequent pancreatic *β*-cell failure, and reduced insulin output, causing a series of complications if left untreated. Unfortunately, the frequency of T2DM is extending globally with various and serious events associated with genes and external factors (Jones et al., 2009; Chatterjee et al., 2017; Manaithiya et al., 2021), so the clinical requirement of finding new targets and drugs to treat T2DM should be given high priority. GPR119 is an orphan G protein-coupled receptor (GPCR) expressed in enteroendocrine cells and pancreatic *β-*cells which signals predominantly through cAMP *via* stimulatory G-protein, and its stimulation by a GPR119 agonist could increase both GLP-1 and insulin secretion (Lauffer et al., 2009; Yang et al., 2018; Xu et al., 2022). Recently, GPR119 has become a research hotspot: it plays an important role in maintenance of glycemic control (Li et al., 2020); it has been considered as a novel therapeutic target of treating dyslipidemia and NASH (Bahirat et al., 2019); and it has emerged as a drug target for T2DM and obesity (Yang et al., 2018). However, no synthetic agonists of GPR119 have been approved as new agents for the treatment of T2DM (Yang et al., 2018; Xu et al., 2022). Therefore, the search for suitable and effective ligands for GPR119 has been ongoing. 3-*O*-MG PIC (**1**), as a GRP119 agonist with a new structural type exhibited strong GPR119 agonism and insulin secretion-stimulating activity at low concentration, suggesting that metabolite **1** was a promising candidate molecule for the treatment of T2DM, and *in vivo* activity is being evaluated. Given the structure disclosure of GPR119-Gs complex (Xu et al., 2022), structure-based drug design, molecular docking, and other computational approaches can be used in future work to carry out structural modification and optimization of **1**.

The yield of **1** under initial transformation conditions was relatively low, which could bring inconvenience to further structural modification and *in vivo* activity evaluation. Optimization of biotransformation conditions is one of the crucial means of increasing yield (Molina et al., 2019; Singh D. et al., 2019). We examined the effects of different pH of medium, culture temperature, rotational speed, and culture duration on the yield of **1**. Significantly, the culture temperature was the most influential factor, and the yield increased dramatically in the range of 24°C to 32°C. It is generally believed that the optimum culture temperature of fungus may be most favorable for its biotransformation process, so the culture and biotransformation temperature of *B. bassiana* was usually 24°C to 28°C in previous work (Xiong et al., 2006; Zhan and Gunatilaka, 2008; Zeng et al., 2010; Sordon et al., 2019; Luzny et al., 2020; Ha et al., 2021). Nonetheless, we found that the growth of *B. bassiana* and the biotransformation efficiency of PIC reached more optimal state under the culture condition of 32°C, which could provide reference for the biotransformation conditions of other substrates by *B. bassiana*. Though the yield of **1** was increased by 4.2-fold compared with that of the control (45.53% vs. 10.09%), almost no substrate was left after the single factor optimization, and the selectivity improvement and large-scale production of **1** are ongoing.

## 5. Conclusion

In summary, we isolated and identified five stilbene mono-methylglucosides (**1**–**5**), including three novel ones (**1–3**), from the fermentation broth by *B. bassiana* transformation after feeding two stilbenes (PIC and RSV), respectively. These results suggest that *B. bassiana* could be applied to obtain the various stilbene *O*-methylglucosides for drug screening by transforming a wide range of stilbenes.

*In vitro* biological test indicated that metabolite 3-*O-*MG PIC (**1**) could activate GPR119 at low concentration of 1 μM, and also significantly promote insulin secretion level, demonstrating that **1** exhibited antidiabetic ability. This finding is useful for the future drug design for more effective and safer ligand targeting GPR119 for the treatment of diabetes.

We further optimized the biotransformation conditions of *B bassiana* to enhance the yield of **1**. After the single factor optimization, the yield of **1** was increased from 10.90% to 45.53%, which lays a foundation for large-scale production of **1**.

## Data availability statement

The original contributions presented in the study are included in the article/Supplementary material, further inquiries can be directed to the corresponding authors.

## Author contributions

TG and PZ are responsible for the overall arrangements for the study. YP and TG designed the biotransformation experiments and drafted the manuscript. YH and LL contributed to bioactivity test. YP and YH performed the substantial experiments. J-JC, T-JC, LL, J-LY, and Z-FS made contributions to data analysis. PZ supervised the study and revised the manuscript. All authors contributed to the article and approved the submitted version.

## Funding

This work was supported by the National Key Research and Development Program of China (grant nos. 2020YFA0908003 and 2018YFA0901900), CAMS Innovation Fund for Medical Sciences (nos. CIFMS2021-I2M-1-029 and 2022-I2M-2-002), and National Natural Science Foundation of China (81803597 and 82200883).

## Conflict of interest

The authors declare that the research was conducted in the absence of any commercial or financial relationships that could be construed as a potential conflict of interest.

## Publisher’s note

All claims expressed in this article are solely those of the authors and do not necessarily represent those of their affiliated organizations, or those of the publisher, the editors and the reviewers. Any product that may be evaluated in this article, or claim that may be made by its manufacturer, is not guaranteed or endorsed by the publisher.
