# Supplementary material for: Microbial biotransformation to obtain stilbene methylglucoside with GPR119 agonistic activity
Source: Front Microbiol. 2023 Mar 22;14:1148513. doi: 10.3389/fmicb.2023.1148513 (PMC10081513; doi:10.3389/fmicb.2023.1148513)
Supplement: Supplementary file 1 [file Data_Sheet_1.docx]

Supplementary Material

**Microbial biotransformation to obtain stilbene methylglucoside with GPR119 agonistic activity**

**Yu Peng^†^, Yi Huan^†^, Jing-Jing Chen, Tian-Jiao Chen, Lei Lei, Jin-Ling Yang, Zhu-Fang Shen, Ting Gong*, Ping Zhu***

***Correspondence:**

Ting Gong

[gongting@imm.ac.cn](mailto:gongting@imm.ac.cn)

Ping Zhu

[zhuping@imm.ac.cn](mailto:gongting@imm.ac.cn)

# Supplementary Figures

**Figure S1.** HRESIMS data of compound **1**.

**Figure S2.** The UV spectrum of compound **1** in CH_3_OH.

**Figure S3.** The IR spectrum of compound **1**.

**Figure S4.** ^1^H NMR spectrum of **1** in CD_3_OD (500 MHz).

**Figure S5.** ^13^C NMR spectrum of **1** in CD_3_OD (125 MHz).

**Figure S6.** HMBC spectrum of **1** in CD_3_OD (500 MHz).

**Figure S7.** HRESIMS data of compound **2**.

**Figure S8.** The UV spectrum of compound **2** in CH_3_OH.

**Figure S9.** The IR spectrum of compound **2**.

**Figure S10.** ^1^H NMR spectrum of **2** in CD_3_OD (500 MHz).

**Figure S11.** ^13^C NMR spectrum of **2** in CD_3_OD (125 MHz).

**Figure S12.** HMBC spectrum of **2** in CD_3_OD (500 MHz).

**Figure S13.** HRESIMS data of compound **3**.

**Figure S14.** The UV spectrum of compound **3** in CH_3_OH.

**Figure S15.** The IR spectrum of compound **3**.

**Figure S16.** ^1^H NMR spectrum of **3** in CD_3_OD (500 MHz).

**Figure S17.** ^13^C NMR spectrum of **3** in CD_3_OD (125 MHz).

**Figure S18.** HMBC spectrum of **3** in CD_3_OD (500 MHz).

**Figure S19.** ^1^H NMR spectrum of **4** in CD_3_OD (500 MHz).

**Figure S20.** ^1^H NMR spectrum of **5** in CD_3_OD (500 MHz).


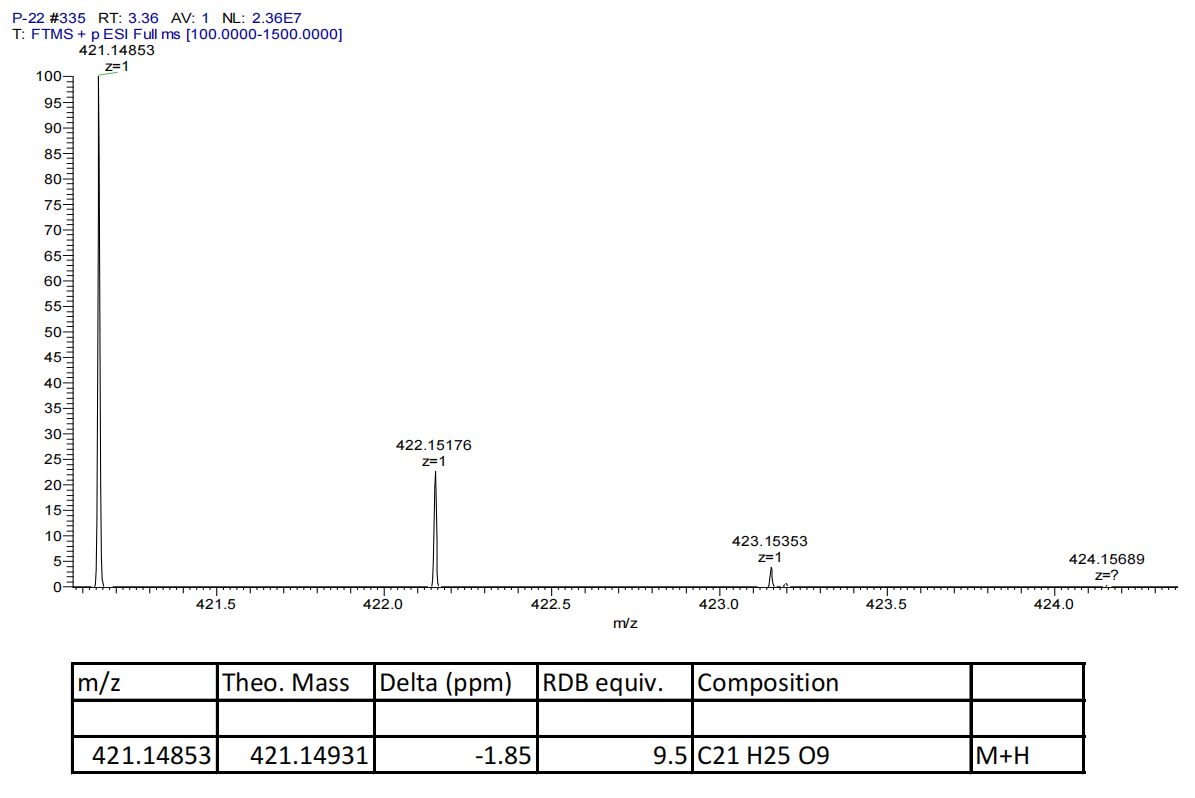

**Figure S1.** HRESIMS data of compound **1**


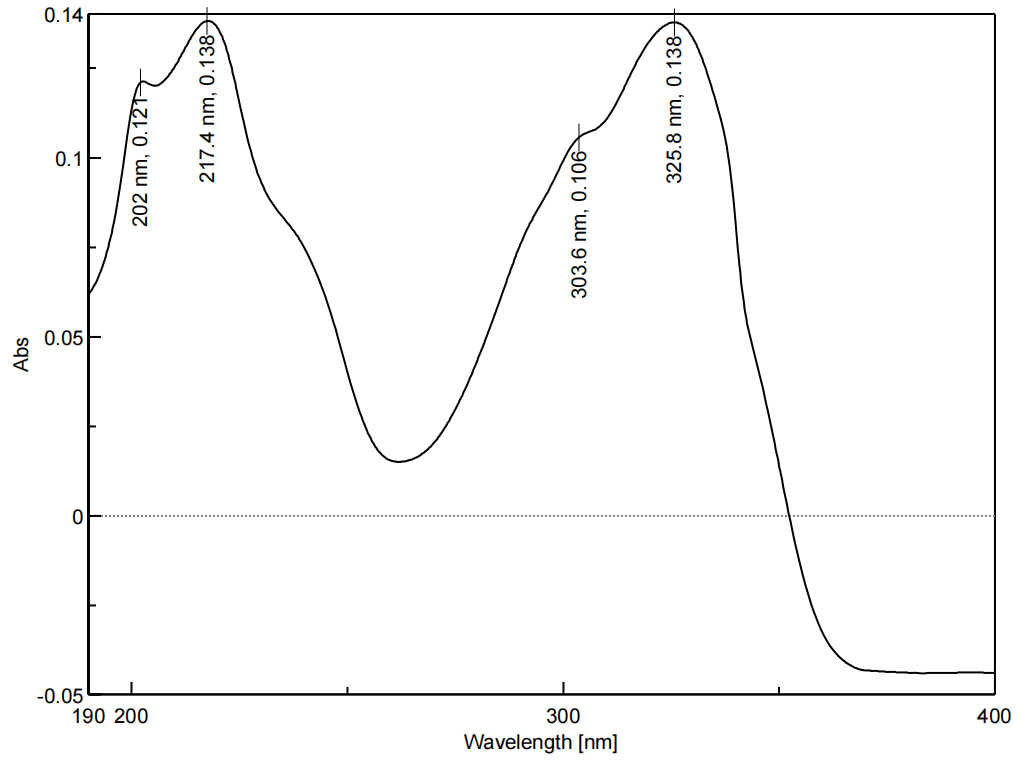

**Figure S2.** The UV spectrum of compound **1** in CH_3_OH


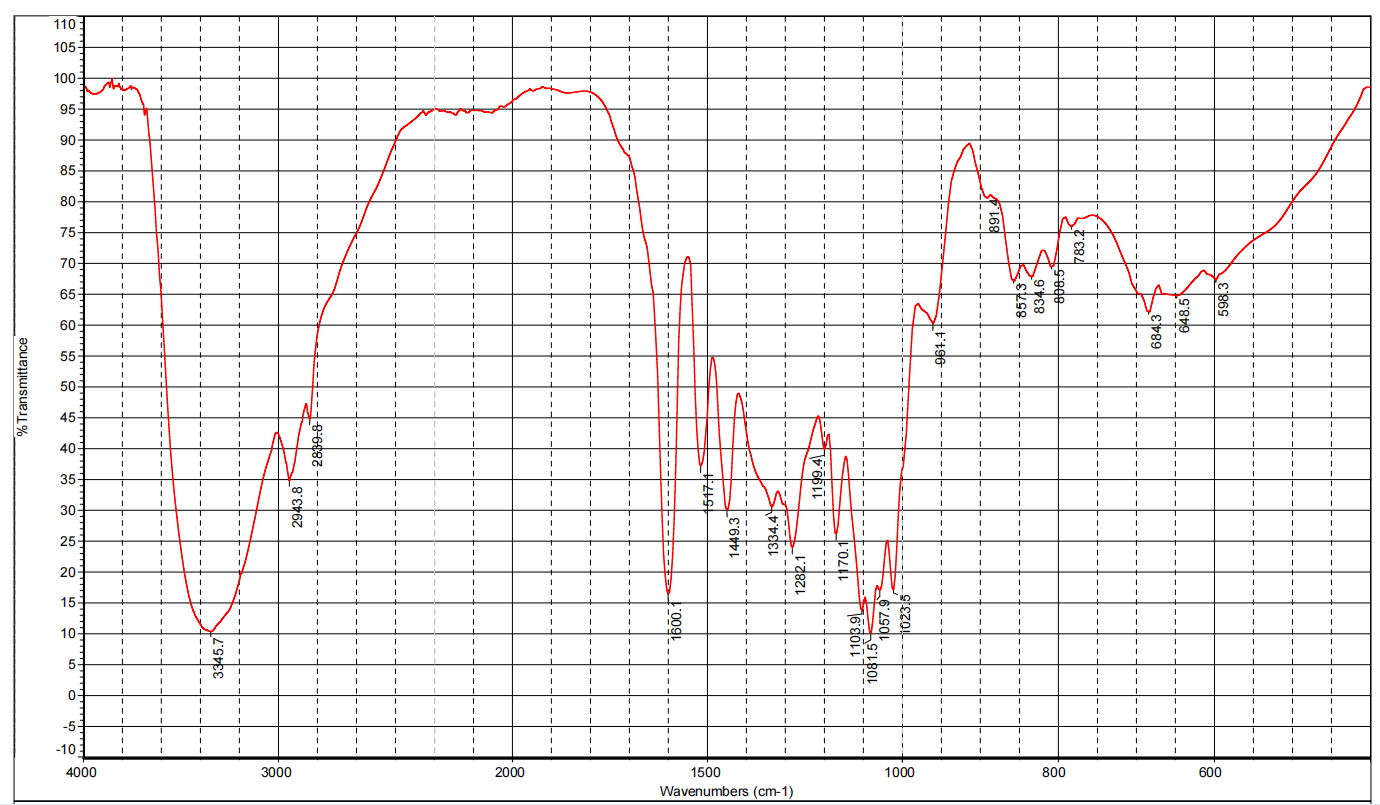

**Figure S3.** The IR spectrum of compound **1**

**Figure S4.** ^1^H NMR spectrum of **1** in CD_3_OD (500 MHz)

**Figure S5.** ^13^C NMR spectrum of **1** in CD_3_OD (125 MHz)

**Figure S6.** HMBC spectrum of **1** in CD_3_OD (500 MHz)


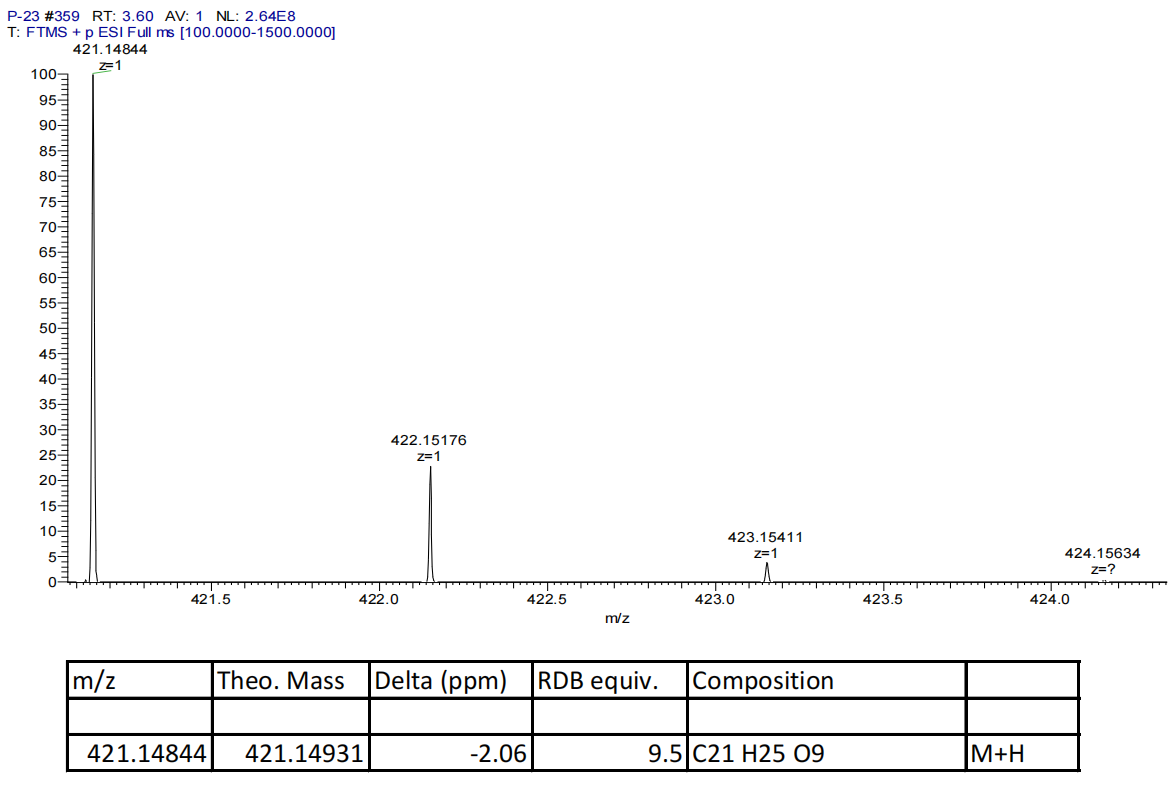

**Figure S7.** HRESIMS data of compound **2**


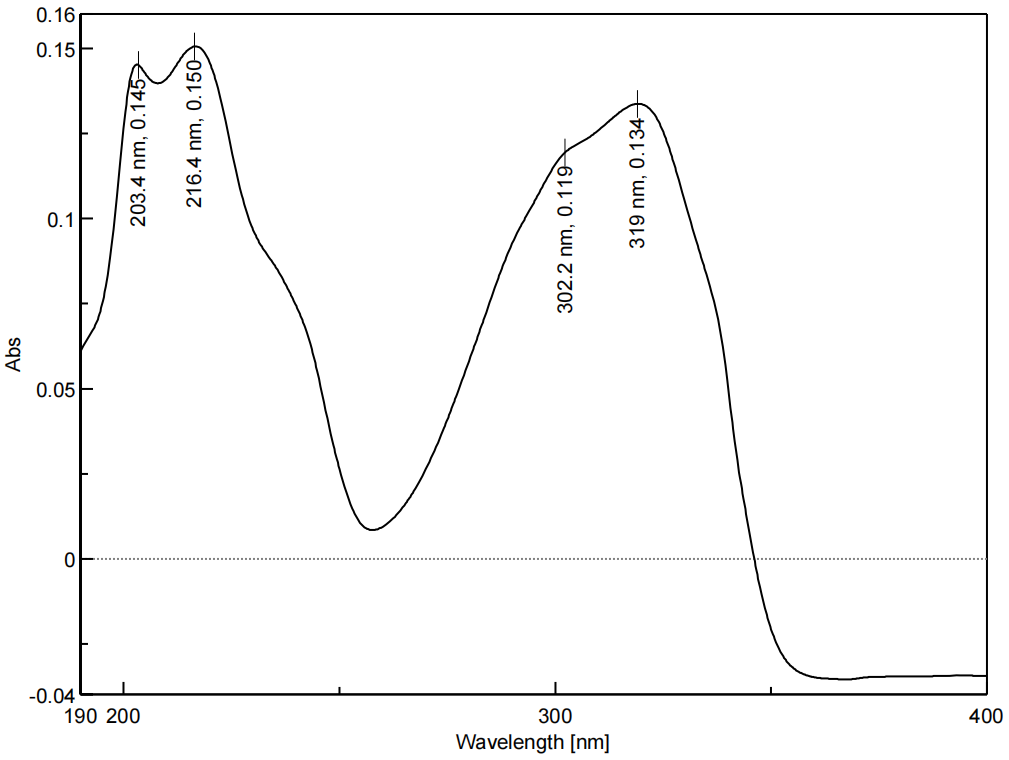

**Figure S8.** The UV spectrum of compound **2** in CH_3_OH


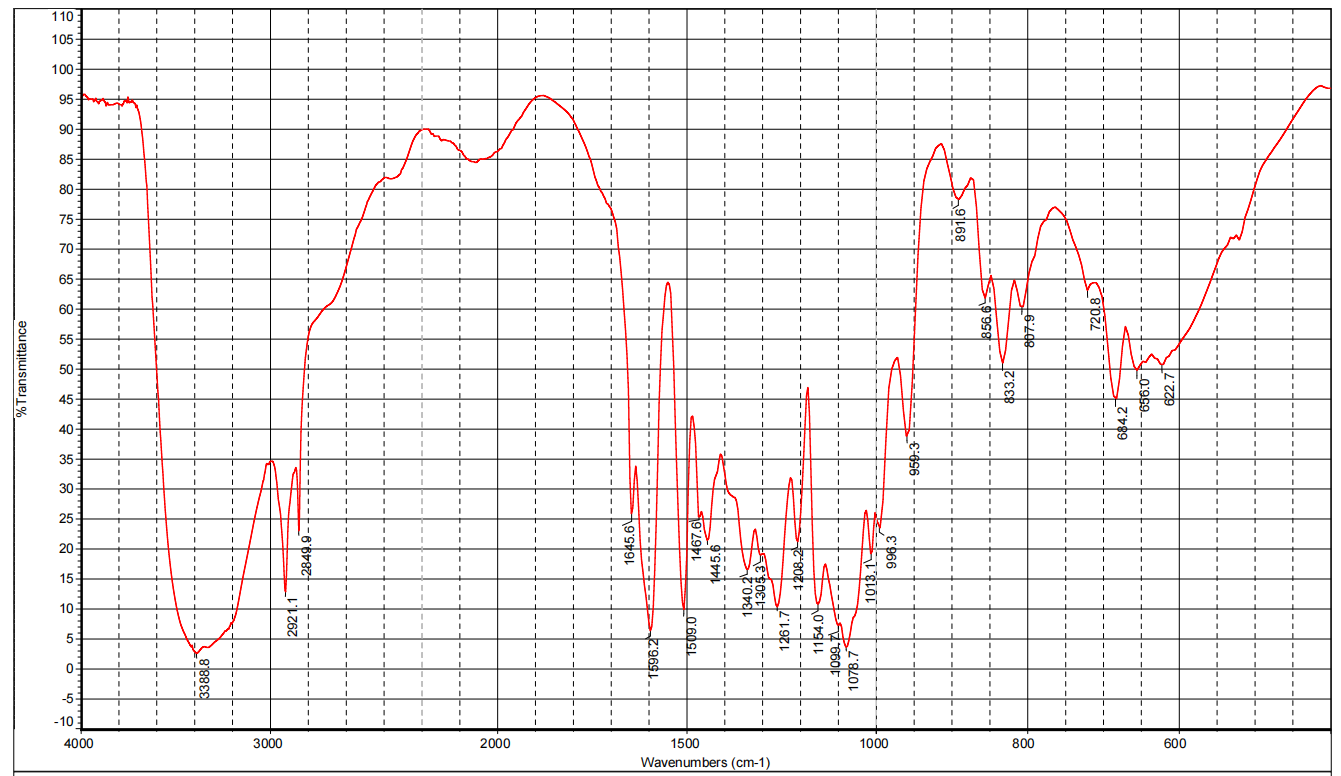

**Figure S9.** The IR spectrum of compound **2**

**Figure S10.** ^1^H NMR spectrum of **2** in CD_3_OD (500 MHz)

**Figure S11.** ^13^C NMR spectrum of **2** in CD_3_OD (125 MHz)

**Figure S12.** HMBC spectrum of **2** in CD_3_OD (500 MHz)


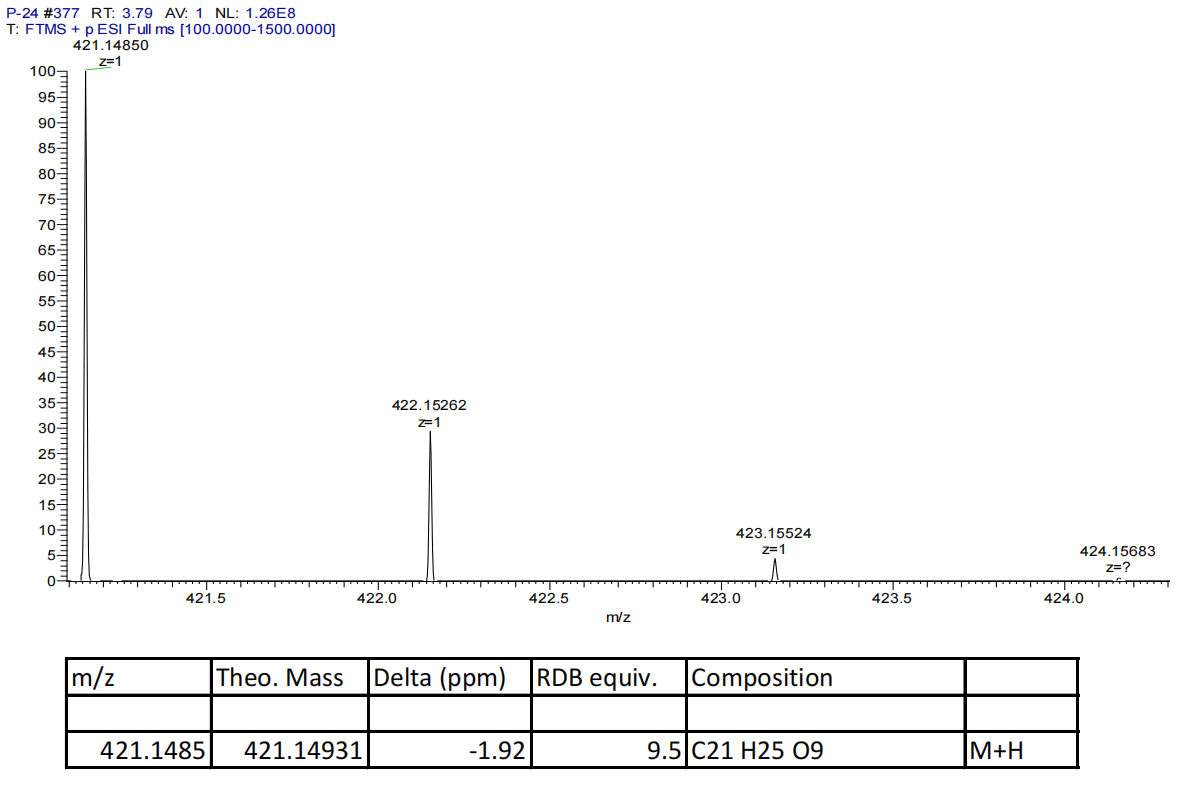

**Figure S13.** HRESIMS data of compound **3**


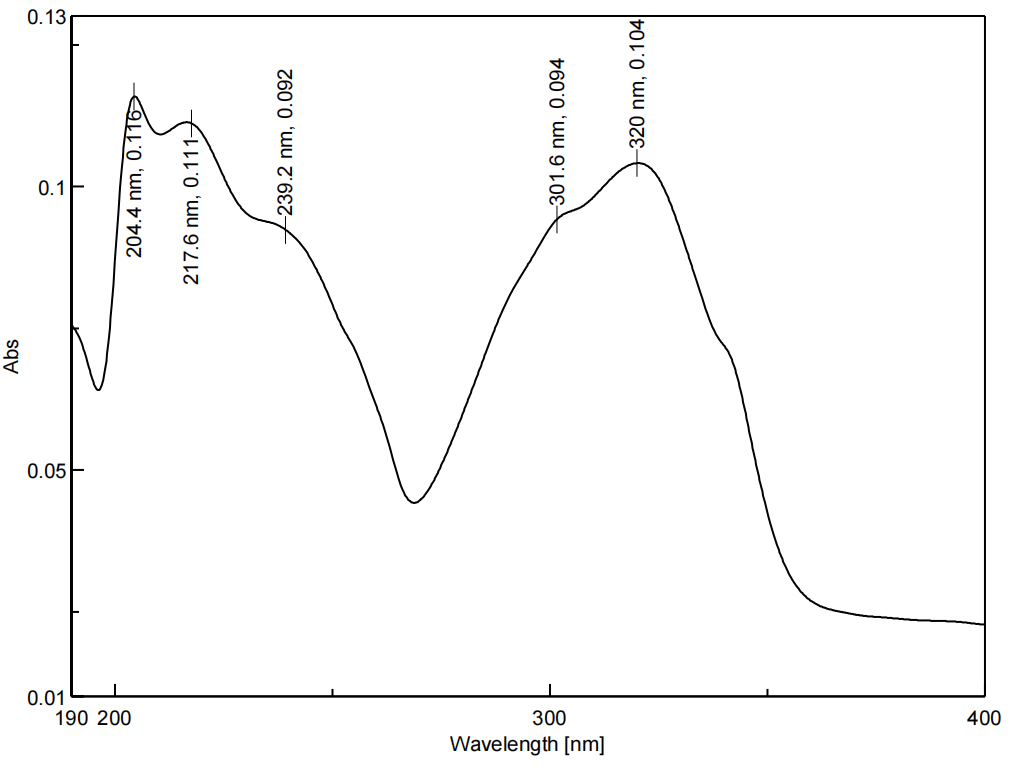

**Figure S14.** The UV spectrum of compound **3** in CH_3_OH


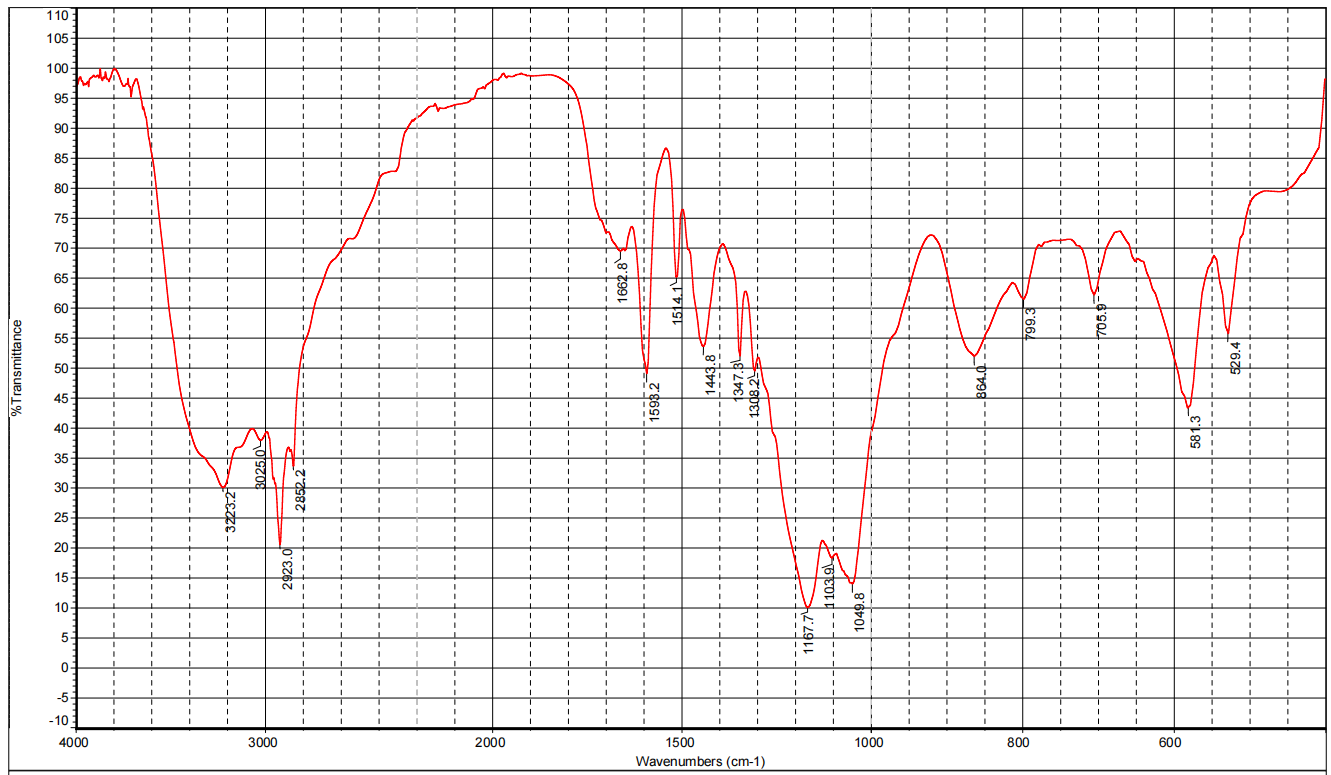

**Figure S15.** The IR spectrum of compound **3**

**Figure S16.** ^1^H NMR spectrum of **3** in CD_3_OD (500 MHz)

**Figure S17.** ^13^C NMR spectrum of **3** in CD_3_OD (125 MHz)

**Figure S18.** HMBC spectrum of **3** in CD_3_OD (500 MHz)


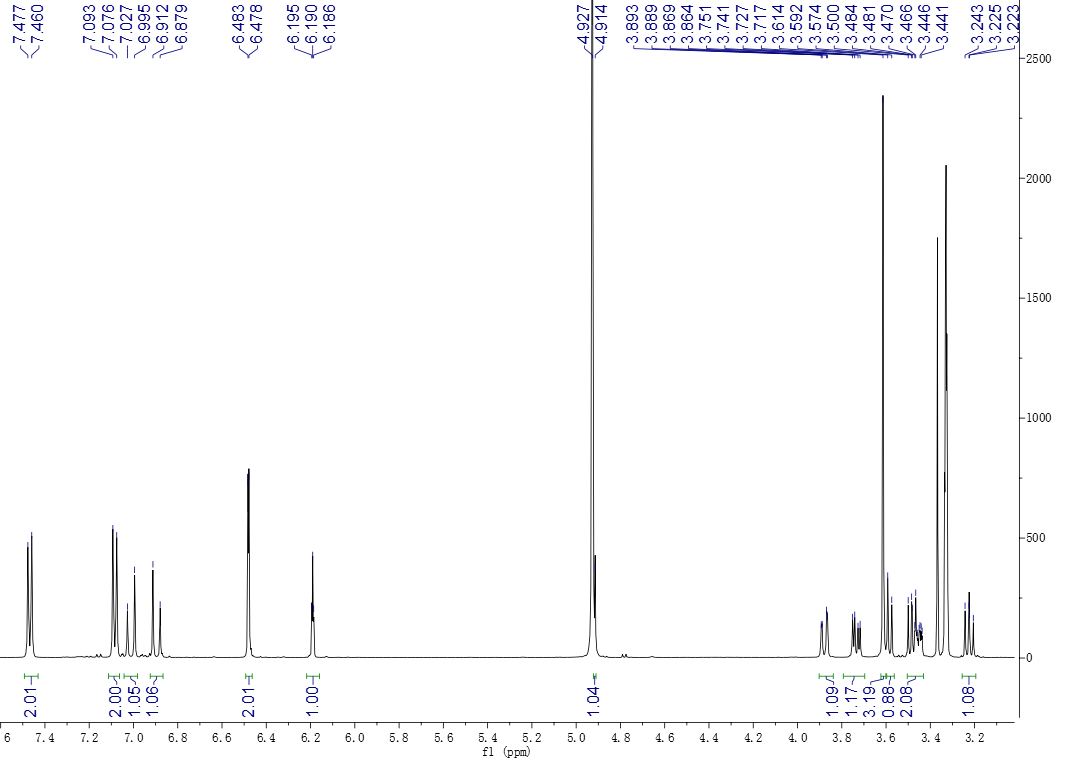

**Figure S19.** ^1^H NMR spectrum of **4** in CD_3_OD (500 MHz)


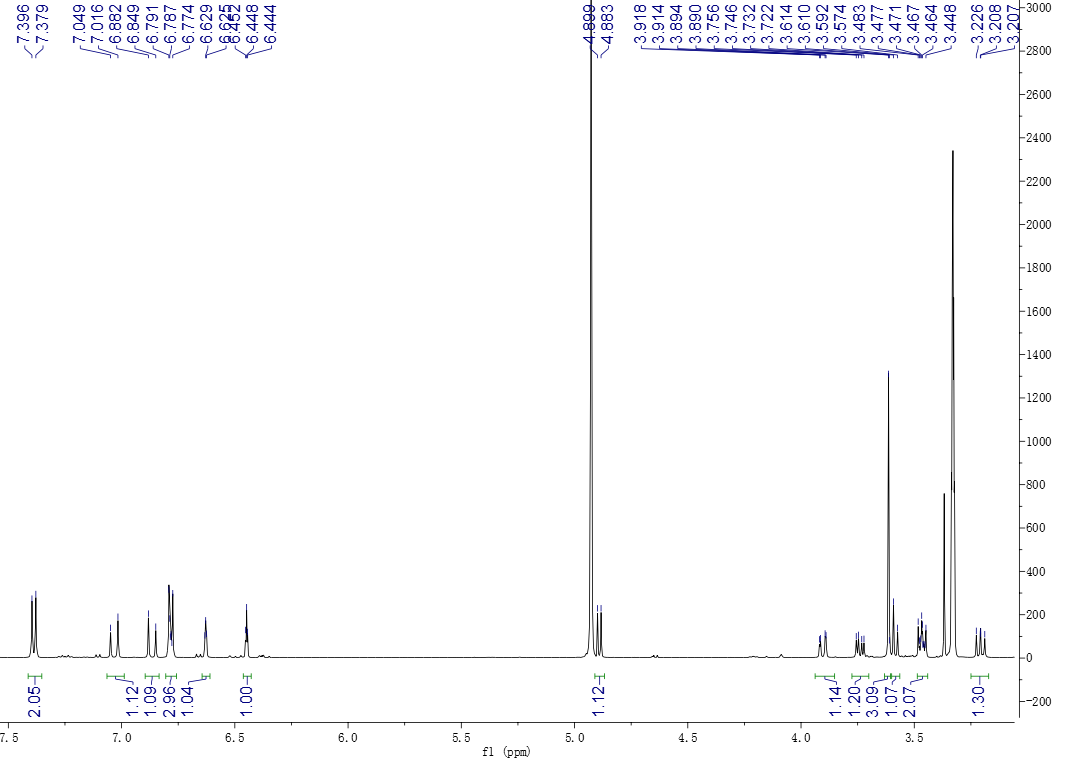

**Figure S20.** ^1^H NMR spectrum of **5** in CD_3_OD (500 MHz)
